# GB-algebraic class

**DOI:** 10.1371/journal.pone.0362407

**Published:** 2026-11-13

**Authors:** Miao Liu, Nueraminaimu Maihemuti, Jiayin Peng, Jiangang Tang

**Affiliations:** 1 School of Mathematics and Statistics, Yili Normal University, Yining, Xinjiang, China; 2 Institute of Applied Mathematics, Yili Normal University, Yining, Xinjiang, China; 3 School of Mathematics and Statistics, Kashi University, Kashi, Xinjiang, China; Northeast Normal University, CHINA

## Abstract

The purpose of this article is to study the relationships among the BCI-algebraic class, the GB-algebraic class, and the class of algebras of type (2,0). The results indicate that: (1) The BCH-algebraic class and the GB-algebraic class are two distinct algebraic classes, both of which contain the BCI-algebraic class. (2) There exist a proper BCH-algebra and a proper GB-algebra, and necessary and sufficient conditions are provided for BCH-algebras and GB-algebras to be proper BCH-algebras and proper GB-algebras, respectively. (3) All (2,0)-type algebras form a proper class, and the GB-algebraic class is a proper subclass of the class of algebras of type (2,0). Several conditions for (2,0)-type algebras to be GB-algebras are given.

## 1 Introduction and preliminaries

The study of BCK-algebras and BCI-algebras can be traced back to the pioneering work of Y. Imai and K. Is’eki in 1966, when they investigated axiom systems of propositional calculi and related algebraic structures [1,2]. These works laid the foundation for the subsequent development of BCK- and BCI-algebra theory, and the theory of BCI-algebras was further developed explicitly by Is’eki in 1980 [3]. In the classical framework, every BCK-algebra is a BCI-algebra, whereas the converse does not hold in general [4,5]. During the 1970s and the early 1980s, considerable attention was devoted to the fundamental properties of BCK-algebras. The basic theory and ideal structures of BCK-algebras were systematically investigated in [5–7], while commutative BCK-algebras and their axiomatic properties were studied in [8–10]. Further investigations concerned tree representations of commutative BCK-algebras [11] and bounded or bounded commutative BCK-algebras [12,13]. From the 1980s onward, research on BCI-algebras gradually expanded in several directions. Quasi-commutative BCI-algebras, examples of BCI-algebras, and associatable BCI-algebras were studied in [14–16], while questions concerning BCI-algebraic classes and *P*-roots were considered in [17,18]. Ideal theory and related structural aspects were further developed through the study of *q*-ideals, *a*-ideals, subalgebras, congruences, and closed ideals [19–21]. Connections between BCI-algebras and Abelian groups, semigroups, and adjoint monoids were investigated in [22–24], whereas topological and normed BCI-algebras were studied in [25,26]. More recently, neutrosophic, hesitant fuzzy, fuzzy, and lattice-valued approaches have further enriched the theory of BCK/BCI-algebras [27–30], and Boolean representations of bounded BCK-algebras have also been investigated [31]. Systematic treatments of BCK- and BCI-algebras can be found in the monographs [4,32,33]. These developments demonstrate that BCK/BCI-algebra theory is closely related to several branches of mathematics, including general algebra, group and semigroup theory, topology, lattice theory, fuzzy set theory, and mathematical logic.

In 1983, Hu and Li introduced the notion of a BCH-algebra as a generalization of BCI-algebras and established its fundamental properties [34]. They subsequently investigated proper BCH-algebras and provided corresponding examples [35]. Since then, the theory of BCH-algebras has been developed from several perspectives. Various types of ideals and their properties were studied in [36,37], while the relationship between BCH-algebras and generalized associative BCI-algebras was investigated in [38]. Fuzzy ideals and fuzzy H-ideals of BCH-algebras were considered in [39], and Peng further introduced closed fuzzy ideals and related fuzzy structures and investigated their fundamental properties and relationships [40]. More recently, structural aspects concerning the BCHK-part of BCH-algebras have also been studied [41].

In 1987, Zhou introduced the notion of GB-algebras and established several of their fundamental properties [42]. Subsequently, the concepts of subalgebras, ideals, and ideal subalgebras of GB-algebras were investigated, together with their basic relationships [43]. In the following year, Zhou further studied homomorphisms and isomorphisms of GB-algebras and obtained several results concerning subalgebras, ideals, quotient algebras, homomorphisms, and isomorphisms [44]. Together with the preceding studies on BCH-algebras, these developments naturally lead to a comparison of BCH-algebras and GB-algebras. Since both can be regarded as generalizations of BCI-algebras, a natural question is what relationship exists between them. More specifically, do the BCH-algebraic class and the GB-algebraic class coincide, or is one contained in the other? Beyond this central question, several related problems naturally arise: Do proper BCH-algebras and proper GB-algebras exist? For every cardinal number ℵ>0, does there exist a GB-algebra of cardinality ℵ? Does the class of all GB-algebras form a proper class? Furthermore, what is the relationship between the GB-algebraic class and the class of all algebras of type (2,0)? The purpose of this paper is to address these questions systematically. We first investigate the relationships among BCI-, BCH-, and GB-algebraic classes and establish necessary and sufficient conditions for BCH-algebras and GB-algebras to be proper. We then construct GB-algebras of arbitrary nonzero cardinality and show that the class of all GB-algebras forms a proper class. Finally, we investigate the relationship between the GB-algebraic class and the class of all algebras of type (2,0) and provide several sufficient conditions under which an algebra of type (2,0) is a GB-algebra.

For convenience, we recall the following standard definitions.

**Definition 1** By a BCI-algebra, we mean a structure (X;*,0), where 0 is a distinguished element and * is a binary operation on *X*, satisfying the following conditions for all a,b,c∈X [3,4]:

(I1) ((a*b)*(a*c))*(c*b)=0;

(I2) (a*(a*b))*b=0;

(I3) a*a=0;

(I4) a*b=0 and b*a=0 imply *a* = *b*;

(I5) a*0=0 implies *a* = 0.

**Definition 2** An algebra (X;*,0) of type (2,0) is called a BCH-algebra if it satisfies the following conditions for all a,b,c∈X [34]:

(H1) a*a=0;

(H2) a*b=0 and b*a=0 imply *a* = *b*;

(H3) (a*b)*c=(a*c)*b.

**Definition 3** An algebra (X;*,0) of type (2,0) is called a GB-algebra if it satisfies the following conditions for all a,b,c∈X [42]:

(G1) a*0=a;

(G2) a*b=0 and b*a=0 imply *a* = *b*;

(G3) ((a*c)*(b*c))*(a*b)=0.

The set *X* in the above three definitions is referred to as the base set of the corresponding algebra.

## 2 GB-algebraic class

To establish the relationship between BCI algebras, BCH algebras, and GB-algebras, we consider some important properties of BCI algebras and BCH-algebras.

**Proposition 1** Suppose (X;*,0) is a BCI-algebra. For a,b∈X, define a≤b if and only if a*b=0. Then the following properties hold:

(P1) a≤b implies c*b≤c*a;

(P2) a≤b and b≤c imply a≤c;

(P3) (a*b)*c=(a*c)*b;

(P4) ((a*c)*(b*c))*(a*b)=0;

(P5) a*b≤c implies a*c≤b;

(P6) a≤b implies a*c≤b*c;

(P7) a*0=a.

**Proof** (P1): If a≤b for a,b∈X, then a*b=0. By (I1), 0=((c*b)*(c*a))*(a*b)=((c*b)*(c*a)) *0, by (I5) we have ((c*b)*(c*a))*0=0 implies (c*b)*(c*a)=0, and so c*b≤c*a. Hence (P1) is true.

(P2): Let a≤b and b≤c for a,b,c∈X. By (P1), a*c≤a*b, since b≤c, and so (a*c)*(a*b)=0. Note that a≤b implies a*b=0, we have (a*c)*0=0. Using (I5), a*c=0, which means that a≤c, and so (P2) holds.

(P3): By (I2) a*(a*c)≤c, which implies (a*b)*c≤(a*b)*(a*(a*c))≤(a*c)*b by (P1) and (I1). Using (P2), (a*b)*c≤(a*c)*b, and so we have (a*c)*b≤(a*b)*c by exchanging *b* and *c*. Using (I4), (a*b)*c=(a*c)*b. Therefore, (P3) is true.

(P4): By (P3) and (I1), ((a*c)*(b*c))*(a*b)=((a*c)*(a*b))*(b*c)=0.

(P5): If a*b≤c, then (a*b)*c=0. By (P3), (a*c)*b=0, and so a*c≤b.

(P6): Let a≤b for a,b∈X, then a*b=0. By (I1), (a*c)*(a*b)≤b*c, using (P5), (a*c)*(b*c)≤a*b, which means that 0=((a*c)*(b*c))*(a*b)=((a*c)*(b*c))*0. By (I5), (a*c)*(b*c)=0. Therefore, a*c≤b*c.

(P7): By (P3) and (I3), (a*0)*a=(a*a)*0=0*0=0. By (I2), (a*(a*0))*0=0, and so a*(a*0)=0 by (I5). Using (I4), we have that (a*0)*a=0 and a*(a*0)=0 imply a*0=a. This completes the proof.

**Proposition 2** If (X;*,0) is a BCH-algebra, then for any a,b∈X, the following hold

(p1) (a*(a*b))*b=0.

(p2) a*0=0 implies *a* = 0.

**Proof** Since (X;*,0) is a BCH-algebra, we have (a*(a*b))*b=(a*b)*(a*b)=0 by (H3) and (H1), thus (p1) holds. If a*0=0, then 0*a=(a*0)*a=(a*a)*0=0*0=0 by (H3) and (H1). Using (H2), we have that a*0=0 and 0*a=0 imply *a* = 0. Therefore, (p2) is true, completing the proof.

**Proposition 3** If (X;*,0) is a GB-algebra, then for any a,b∈X, the following hold

(p-1) a*a=0.

(p-2) a*0=0 implies *a* = 0.

**Proof** Since (X;*,0) is a GB-algebra, we have a*a=((a*0)*(0*0))*(a*0)=0 by (G1) and (G3), thus (p-1) is true. If a*0=0, then 0*a=(a*0)*a=a*a=0*0=0 by (G1) and (p-1). Using (G2), we have that a*0=0 and 0*a=0 imply *a* = 0. Therefore, (p-2) holds, completing the proof.

**Theorem 1** Any BCI-algebra is both a BCH-algebra and a GB-algebra.

**Proof** Let (X;*,0) be a BCI-algebra, then the establishment of (I3) and (I4) implies the establishment of (H1) and (H2), respectively. By (P3), (H3) holds. Hence, (X;*,0) is a BCH-algebra. By (P7), (I4) and (P4), we have that (G1), (G2) and (G3) hold. Therefore, (X;*,0) is a GB-algebra, completing the proof.

For convenience, we introduce the following definitions.

**Definition 4** A class composed of all BCI-algebras (BCH-algebras, GB-algebras) is called BCI-algebraic (BCH-algebraic, GB-algebraic) class.

**Definition 5** If a BCH-algebra (GB-algebra) is not a BCI-algebra, it is called a proper BCH-algebra (GB-algebra).

According to Theorem 1, we have the following: BCI-algebraic class ⊆ BCH-algebraic class and BCI-algebraic class ⊆ GB-algebraic class.

Now, let’s consider the relationship between BCH-algebras and GB-algebras. By comparing Definitions 2 and 3, we see that condition (H2) is identical to condition (G2). If (X;*,0) is a BCH-algebra, by (H3) and (H1), (a*0)*a=(a*a)*0=0*0=0, and (a*(a*0))*0=(a*0)*(a*0)=0 which implies a*(a*0)=0 by (p2). By (H2), we have that (a*0)*a=0 and a*(a*0)=0 imply a*0=a, and so (G1) holds.

Conversely, if (X;*,0) is a GB-algebra, by (G3) and (G1), 0=((a*0)*(0*0))*(a*0)=(a*0)*(a*0)=a*a, thus (H1) is true.

**Example 1** Let *X* = {0, 1, 2, 3}, the operation * is given by the following table



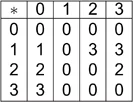

(1)


then (X;*,0) is a proper BCH-algebra. In fact, it is easy to verify that (X;*,0) is a BCH-algebra, but ((2*3)*(2*1))*(1*3)=(2*0)*3=2*3=2≠0, and so ((2*3)*(1*3))*(2*1)=2≠0, which means that neither (I1) nor (G3) is valid. Therefore, (X;*,0) is neither a BCI-algebra nor a GB-algebra, and so (X;*,0) is a proper BCH-algebra.

**Example 2** Suppose *X* = {0, 1, 2, 3}, the operation * is given by the table



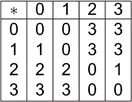

(2)


then (X;*,0) is a proper GB-algebra. In fact, by calculation, we can easily verify that (X;*,0) is a GB-algebra, but (2*(2*3))*3=(2*1)*3=2*3=1≠0 and (2*3)*1=0≠1=(2*1)*3. That is, neither (I2) nor (H3) is valid. Thus, (X;*,0) is neither a BCI-algebra nor a BCH-algebra, and so (X;*,0) is a proper GB-algebra.

From the discussion of Example 1 and Example 2, the following conclusions can be drawn:

**Theorem 2** There exist a proper BCH-algebra and a proper GB-algebra, and the BCH-algebraic class and the GB-algebraic class are distinct.

**Theorem 3** (i) a BCH-algebra (X;*,0) is a proper BCH-algebra if and only if it does not satisfy condition (I1). (ii) a GB-algebra (X;*,0) is a proper GB-algebra if and only if it does not satisfy condition (H3).

**Proof** (i): Suppose that (X;*,0) is a proper BCH-algebra. If (X;*,0) satisfies condition (I1), then by Proposition 2, conditions (I2) and (I5) hold. Moreover, since (X;*,0) is a BCH-algebra, conditions (I3) and (I4) follow from (H1) and (H2), respectively. Thus, (X;*,0) is a BCI-algebra, which contradicts the assumption that (X;*,0) is a proper BCH-algebra. Therefore, (X;*,0) does not satisfy condition (I1), and hence the necessity holds.

Conversely, suppose that (X;*,0) does not satisfy condition (I1). Then (X;*,0) is not a BCI-algebra. Since (X;*,0) is a BCH-algebra, it follows that (X;*,0) is a proper BCH-algebra. Hence, the sufficiency holds.

(ii): If a GB-algebra (X;*,0) satisfies condition (H3), then ((a*b)*(a*c))*(c*b)=((a*b)*(c*b))*(a*c)=0 by (H3) and (G3), and (a*(a*b))*b=(a*b)*(a*b)=0 by (H3) and (p-1).Thus, (I1) and (I2) hold. Moreover, (I3), (I4), and (I5) follow from (p-1), (G2), and (p-2), respectively. Therefore, (X;*,0) is a BCI-algebra, which contradicts the assumption that (X;*,0) is a proper GB-algebra.

Conversely, assume that (X;*,0) does not satisfy condition (H3). Then (X;*,0) is not a BCI-algebra. Otherwise, (P3) would hold, contradicting the assumption that (H3) does not hold. Therefore, (X;*,0) is a proper GB-algebra.

**Theorem 4** If a GB-algebra (X;*,0) satisfies one of the following conditions,

(1) (x*y)*z=(x*z)*y,

(2) x*y=y*x and (x*y)*z=x*(y*z),

then (X;*,0) is a BCI-algebra.

**Proof** If condition (1) holds, then (a*(a*b))*b=(a*b)*(a*b)=0 by (1) and (p-1), which means that (I2) holds. By (1) and (G3), ((a*b)*(a*c))*(c*b)=((a*b)*(c*b))*(a*c)=0, where the last equality follows from (G3) by taking *x* = *a*, *y* = *c*, and *z* = *b*. Hence, (I1) holds. It follows from (p-1), (G2), and (p-2) that conditions (I3), (I4), and (I5) hold, respectively. Therefore,(X;*,0) is a BCI-algebra.

If condition (2) holds, then (x*y)*z=x*(y*z)=x*(z*y)=(x*z)*y, so condition (1) holds. By the first part of the proof, (X;*,0) is a BCI-algebra.

**Lemma 1** Let *X* be a partially ordered set with a partial order relation of ≤. There is a minimum element of 0 in *X*, but any two non-zero elements in *X* cannot be compared under relation ≤. Define the operation * in *X* as follows: for any x,y∈X,


$$\begin{array}{c} x*y=\left\{ \begin{array}{cc} \text{0}, & x\leq y;\\ x, & \text{otherwise}. \end{array}\right. \end{array}$$ (3)

Then (X;*,0) is a GB-algebra.

**Proof** For any x,y∈X, when *x* = 0, we have x≤y since 0 is a minimum element, and so x*y=0=x. When *y* = 0 and x≠0, we have x*y=x. When x≠0, y≠0 and x≠y, we have x*y=x. When x≠0, y≠0 and *x* = *y*, we have x*y=0. In short, if x=y≠0, we have x*y=0; otherwise, x*y=x.

Clearly,x*0=x, and hence (G1) holds. For any x,y,z∈X, when x=y=z≠0, we have that x*y=0, x*z=0 and y*z=0. Thus, ((x*z)*(y*z))*(x*y)=(0*0)*0=0; when x=y∈X−{0,z}, we get that x*z=x and y*z=y. Hence ((x*z)*(y*z))*(x*y)=(x*y)*(x*y)=0; when x=z∈X−{0,y}, we have ((x*z)*(y*z))*(x*y)=(0*(y*z))*(x*y)=0*(x*y)=0; when y=z∈X−{0,x}, we have ((x*z)*(y*z))*(x*y)=(x*0)*x=x*x=0; Otherwise, ((x*z)*(y*z))*(x*y)=(x*y)*(x*y)=0. Therefore, ((x*z)*(y*z))*(x*y)=0 for all x,y,z∈X, which means that (G3) holds. Let x*y=0 and y*x=0. Assume x≠y, we have that x=x*y=0=y*x=y, which is contradictory. Therefore (G2) holds. According to the above analysis, (X;*,0) is a GB-algebra.

**Lemma 2** For any non-empty set *X*, a GB-algebra can be constructed, whose base set is *X*.

**Proof** Because *X* is a non-empty set, we can take any element x∈X and denote it as 0. If *X* contains only the element 0, i.e., *X* = {0}, then define the operation * in *X* as: 0*0=0. It is easy to verify that (X;*,0) is a GB-algebra and *X* is its base set.

Generally, we first establish a partial order relationship ≤ in *X*: for any b∈X, 0≤b and b≤b; For any two different non-zero elements a,b∈X, *a* and *b* cannot be compared. It is easy to verify that (X,≤) is a partially ordered set. Define a binary operation * in *X* that is the same as Eq. (3), then according to Lemma 1, (X;*,0) is a GB-algebra.

**Theorem 5** For every cardinal number ℵ>0, there exists a GB-algebra of cardinality ℵ.

**Proof** Let ℵ>0 be an arbitrary cardinal number. Identifying cardinals with initial ordinals, let X={α:α<ℵ}. Then |X|=ℵ [45,46]. Choose the ordinal 0 as the distinguished element of *X* and define the binary operation * on *X* as in Eq. (3). By Lemma 1 and Lemma 2, (X;*,0) is a GB-algebra. Therefore, there exists a GB-algebra of cardinality ℵ.

**Corollary 1** For every positive integer *n*, there exists a GB-algebra with *n* elements.

It is worth mentioning that the proof of Theorem 5, based on Lemma 1 and Lemma 2, provides a method for constructing a GB-algebra of every nonzero cardinality. For each cardinal ℵ>0, denote such a GB-algebra by $X_{ℵ}$. If ℵ≠κ, then $|X_{ℵ}|=ℵ\text{≠}\kappa =|X_{\kappa }|,$ and hence $X_{ℵ}\text{≠}X_{\kappa }$. Since there exists a distinct GB-algebra for every nonzero cardinal and the class of all cardinals is a proper class, the class of all GB-algebras cannot be a set.Therefore, we have

**Corollary 2** All GB-algebras form a proper class.

We provide an example of obtaining a GB-algebra using the above construction method.

**Example 3** Suppose *X* = {0, 1, 2, 3, 4, 5}, the operation * in *X* is given by the table



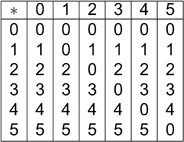

(4)


Then (X;*,0) is a GB-algebra with cardinality 6.

**Theorem 6** All (2,0)-type algebras form a proper class (called a (2,0)-type algebraic class). The GB-algebraic class is a proper subclass of the (2,0)-type algebraic class.

**Proof** Every GB-algebra is an algebra of type (2,0), and hence the GB-algebraic class is contained in the class of all algebras of type (2,0). By Corollary 2, the class of all GB-algebras is a proper class. Therefore, the class of all algebras of type (2,0) is also a proper class.

To show that the GB-algebraic class is a proper subclass of the (2,0)-type algebraic class, consider the following structure (X;*,0), where *X* = {0,1} and the binary operation * is defined by the following table:



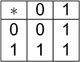

(5)


Then (X;*,0) is a (2,0)-type algebra, but it is not a GB-algebra since ((1*0)*(0*0))*(1*0)=(1*0)*1=1*1=1≠0. Therefore, the GB-algebraic class is a proper subclass of the (2,0)-type algebraic class.

From Theorem 3, if a GB-algebra satisfies condition (H3), then it is a BCI-algebra. Similarly, we have

**Theorem 7** If a (2,0)-type algebra (X;*,0) satisfies (G1), (G2), (p-1) and for all a,b,c∈X,


$$\begin{array}{c} a*b=b*a,\;(a*b)*c=a*(b*c) \end{array}$$ (6)

then (X;*,0) is a GB-algebra.

**Proof** It remains only to verify condition (G3). For any a,b,c∈X,


$$\begin{array}{cc} \left((a*c)*(b*c))*(a*b\right) & =((a*c)*(c*b))*(a*b)\\ & =(((a*c)*c)*b)*(a*b)\\ & =((a*(c*c))*b)*(a*b)\\ & =((a*0)*b)*(a*b)\\ & =(a*b)*(a*b)\\ & =0. \end{array}$$ (7)

Therefore, (G3) holds. Together with (G1) and (G2), it follows that (X;*,0) is a GB-algebra.

Note that Eq. (6) states that the operation * is commutative and associative.

**Theorem 8** If a (2,0)-type algebra (X;*,0) with an associative binary operation satisfies (G1), (G2) and for any x,y,z∈X,


$$\begin{array}{c} (x*(y*z))*((y*x)*z)=0. \end{array}$$ (8)

Then (X;*,0) is a GB-algebra.

**Proof** Since (X;*,0) satisfies (G1) and (G2), it remains only to verify condition (G3). For any a,b,c∈X, by associativity, we have


$$\begin{array}{cc} \left((a*c)*(b*c))*(a*b\right) & =(((a*c)*b)*c)*(a*b)\\ & =((a*c)*b)*(c*(a*b))\\ & =(a*(c*b))*((c*a)*b)\\ & =0. \end{array}$$ (9)

The last equality follows from condition (8) by taking *x* = *a*, *y* = *c*, and *z* = *b*. Therefore, (G3) holds. Together with (G1) and (G2), it follows that (X;*,0) is a GB-algebra.

**Theorem 9** If a (2,0)-type algebra (X;*,0) with an associative binary operation satisfies (G1), (G2), (p-1), and for any x,y,z∈X,


$$\begin{array}{c} (x*y)*(x*z)=y*z, \end{array}$$ (10)

then (X;*,0) is a GB-algebra.

**Proof** Since (X;*,0) satisfies (G1) and (G2), it remains only to verify condition (G3). For any a,b,c∈X, by associativity and condition (10), we have


$$\begin{array}{cc} \left((a*c)*(b*c))*(a*b\right) & =(((a*c)*b)*c)*(a*b)\\ & =((a*c)*b)*(c*(a*b))\\ & =(a*(c*b))*((c*a)*b)\\ & =((a*(c*b))*(c*a))*b\\ & =(a*((c*b)*(c*a)))*b\\ & =(a*(b*a))*b\\ & =a*((b*a)*b)\\ & =a*(b*(a*b))\\ & =(a*b)*(a*b)\\ & =0. \end{array}$$ (11)

Here, the equality (c*b)*(c*a)=b*a follows from condition (10) by taking *x* = *c*, *y* = *b*, and *z* = *a*. The last equality follows from (p-1). Therefore, (G3) holds. Together with (G1) and (G2), it follows that (X;*,0) is a GB-algebra.
